# Cu^2^^+^ Modulates Enzymatic Browning in Potato Tubers Through Amino Acid and Organic Acid Metabolism

**DOI:** 10.3390/foods14223816

**Published:** 2025-11-07

**Authors:** Shulei Feng, Jinli Li, Rong Guo, Lixiang Cheng, Gang Sa, Jianlong Yuan, Hongyu Yang, Juan Liu, Bin Yu

**Affiliations:** 1 State Key Laboratory of Aridland Crop Science, Gansu Agricultural University, Lanzhou 730070, China; 1073323120577@st.gsau.edu.cn (S.F.); 1073323120596@st.gsau.edu.cn (J.L.); 1073323120553@st.gsau.edu.cn (R.G.); chenglx@gsau.edu.cn (L.C.); sag@gsau.edu.cn (G.S.); 14334@gsau.edu.cn (J.Y.); 2 College of Agronomy, Gansu Agricultural University, Lanzhou 730070, China; 3 College of Horticulture, Gansu Agricultural University, Lanzhou 730070, China; yanghongyu3969@gsau.edu.cn; 4 College of Water Conservancy and Hydropower Engineering, Gansu Agricultural University, Lanzhou 730070, China

**Keywords:** potato, copper application, polyphenol oxidase, free amino acid metabolism, organic acid, browning index

## Abstract

Enzymatic browning markedly reduces the processing quality and economic value of potatoes. This study evaluated the effects of copper sulfate (CuSO_4_) on potato growth, yield, and enzymatic browning. Quantitative analysis of browning-related enzymes, organic acids, free amino acids, and the browning index (BI) was conducted using high-performance liquid chromatography and non-contact colorimetric technology. Moderate Cu^2+^ (0.078–0.157 mmol·L^−1^) supply enhanced photosynthetic capacity, biomass, and starch accumulation, whereas excessive Cu induced oxidative stress, increased PPO activity, phenolic accumulation, and BI. Metabolic profiling revealed that Cu^2+^ activates PPO via its copper-binding sites and reprograms amino acid and organic acid metabolism—upregulating arginine and proline while downregulating isoleucine, leucine, phenylalanine, lysine, citrate, and chlorogenic acid, all strongly correlated with BI. These findings highlight the dual role of copper in yield formation and enzymatic browning, introducing metabolic reprogramming as a potential mechanism for optimizing Cu fertilization to balance productivity and postharvest quality.

## 1. Introduction

Potato (*Solanum tuberosum* L.) is one of the most important non-grain crops globally [1]. Enzymatic browning is one of the primary challenges faced during potato processing [2]. Mechanical operations such as peeling and slicing disrupt the cell structure, breaking the spatial separation between phenolic substrates and enzymes, which leads to rapid oxidation reactions. This results in darkening of the processed products, deterioration of flavor, significant reduction in sensory quality and commercial value, and nutrient loss [3]. Therefore, effectively controlling enzymatic browning has become an urgent issue that needs to be addressed in the potato processing industry.

Enzymatic browning primarily occurs under aerobic conditions, where oxidases catalyze polyphenolic substrates, generating quinones that further polymerize to form brown melanin [4,5]. Among these, PPO is the most critical rate-limiting enzyme, catalyzing the oxidation of catechol to o-quinones, which can then react with amino acids or proteins, accelerating pigment formation [6]. POD, another important oxidase, catalyzes the oxidation of phenolic compounds to quinones in the presence of hydrogen peroxide, which subsequently polymerize to form brown substances [7]. PAL, a key wound-induced enzyme, exhibits increased activity due to cell damage during processing [8]. Elevated PAL activity promotes the accumulation of phenolic compounds, which serve as substrates for PPO and POD [9].

Recent studies on the regulation of enzymatic browning have increasingly focused on the role of metabolites. Among these, amino acids have been shown to influence PPO activity and substrate accumulation through various mechanisms. Glutamic acid, aspartic acid, proline, isoleucine, and L-cysteine have been found to inhibit browning, while tyrosine, lysine, arginine, and serine exacerbate it [10,11,12,13,14]. The mechanisms underlying these effects involve multiple pathways, such as regulation of enzyme activity, metal chelation, pH modulation, and antioxidant activity. Some amino acids can bind to copper ions in the active center of PPO, altering its conformation and thereby inhibiting its activity [15,16]. Additionally, organic acids are widely used to control browning. Ascorbic acid inhibits browning by reducing the activity of PPO and POD and preventing the oxidation of total phenolic compounds [17]. Citric acid can suppress browning by forming complexes with phenolic substrates or PPO enzymes [18]. Chlorogenic acid directly inhibits PPO activity while maintaining cellular antioxidant status and preserving quality stability [19,20].

Copper ions play a central catalytic role in enzymatic browning, being a key component of the PPO active center [21]. PPO is a copper-containing terminal oxidoreductase encoded by nuclear genes [22]. Its active structure consists of two copper ions (CuA and CuB) and histidine residues [23]. The presence of copper ions not only determines the activity level of PPO but also influences its substrate specificity and catalytic efficiency. Consequently, regulating the interaction between copper ions and PPO has become a significant focus in recent enzymatic browning research. In addition, copper, as an essential trace element for plants, plays a crucial role in photosynthesis, antioxidant metabolism, nitrogen absorption, and protein synthesis. Adequate copper supplementation enhances photosynthetic efficiency, nutrient metabolism, and stress resistance in crops, thereby improving both quality and yield [24,25]. Studies have shown that copper sulfate treatment increases chlorophyll content and antioxidant enzyme activity in potato leaves, delays leaf senescence, and improves biomass accumulation [26].

Although previous studies have made progress in the enzymatic mechanism of enzymatic browning and its partial regulation methods, the research on the interaction between copper ions and plant metabolic network is still weak. Especially in potato, how copper ions synergistically affect the metabolic mechanism of enzymatic browning by regulating multiple pathways such as amino acid metabolism, organic acid accumulation, and enzyme activity status has not been systematically analyzed. Compared with other metal ions such as Fe^2+^ and Zn^2+^, Cu^2+^ possesses stronger redox capacity and higher affinity for polyphenol oxidase (PPO), resulting in distinct effects on enzymatic activity and metabolic balance. Therefore, we hypothesize that copper may exert a concentration-dependent dual regulation on potato tuber physiology and enzymatic browning through coordinated modulation of amino acid and organic acid metabolism.

In this study, the effect of different concentrations of copper sulfate on the regulation of enzymatic browning in potato tubers was investigated using “Xisen No. 6” potatoes as the experimental material. The changes in color difference, browning index, activity of key enzymes (PPO, POD, PAL), total phenolics, malondialdehyde, representative amino acids, and organic acids (ascorbic acid, citric acid, chlorogenic acid), as well as other metabolites, were measured following treatment to reveal the potential mechanism of copper ion intervention in enzymatic browning through the regulation of a multi-level metabolic network. This study enhances our understanding of the physiological role of metal ions in potato quality regulation and provides theoretical support and a technical pathway for developing safe and effective anti-browning strategies.

## 2. Materials and Methods

### 2.1. Test Materials

The main cultivated potato variety in China, “Xisen No. 6”, was used as the test material, provided by the Key Laboratory of Arid Crops Science of Gansu Agricultural University. This variety is characterized by high yield, disease resistance, and wide adaptability, making it suitable for both fresh consumption and starch processing.

#### 2.1.1. Preparation of Test Materials

The tissue-cultured seedlings of Xisen No. 6 were propagated to 300 plants and cultured for 21 days in a tissue culture room with a light intensity of 2000 lx, light cycle of 16L/8D, temperature of 25 ± 1 °C, and relative humidity of 60–70%. After 21 days, the culture containers were opened and placed in a greenhouse (temperature 22–25 °C, natural light), where the seedlings were acclimated for 24 h at room temperature. Healthy and uniform tissue-cultured seedlings were selected, and their root matrices were rinsed with sterile water. The main roots were trimmed to 1 cm in length and transplanted into flowerpots (12 cm diameter, 10 cm height) containing 300 g of vermiculite (pH 7.0–7.5, pre-sterilized at 121 °C for 20 min). One seedling was transplanted into each pot (serving as an independent biological replicate), and the plants were cultured in a greenhouse. After 14 days, the plant height was uniform, with 5–6 leaves per plant. A total of 150 healthy plants (corresponding to 30 biological replicates per treatment) were selected and transferred to an artificial climate chamber (16L/8D, 25 ± 1 °C, relative humidity 65%) for continued growth.

#### 2.1.2. Test Treatment

This experiment utilized a completely randomized block design with a single factor. Based on an improved MS nutrient solution (pH 5.8), five Cu^2+^ concentration gradients were established by adding different levels of CuSO_4_ (pH 5.8, analytical grade, Shanghai Zhanyun Chemical Co., Ltd., Shanghai, China), making the copper ion concentrations 0, 0.078, 0.157, 0.235, and 0.313 mmol·L^−1^, which were labeled as CK, T1, T2, T3, and T4, respectively (Table 1). Each treatment group included 30 biological replicates (one independent potato plant per flowerpots), with the experimental error estimated from independent samples. Additionally, three technical replicates were included for the measurement of indicators such as plant height and biochemical parameters to minimize random errors associated with instrument operation and detection. The sample size of 30 was chosen to accommodate potential sample loss and increase sensitivity for detecting subtle treatment effects. In the early stages of tuber formation, the roots were irrigated with MS basal nutrient solution without Cu^2+^. Each pot received 300 mL of solution once every 3 days for 2 consecutive applications to ensure balanced plant nutrition. After 7 days, treatments began, with 300 mL applied once every 7 days for a total of 5 applications. The water content of the vermiculite was monitored in real-time using a TDR350 soil moisture meter (Spectrum Technologies, Plainfield, IL, USA) and maintained at 70–75% (*w*/*w*).

#### 2.1.3. Sampling

At the flowering stage, three plants exhibiting robust growth and consistent phenotypes were randomly selected from each treatment to measure plant height and stem diameter (each measurement was performed in 3 technical replicates to reduce instrumental error). Leaf and tuber samples were collected at the mature tuber stage (when over 90% of the plants had naturally withered, and the stems and leaves completely lost their green color and began lodging) for various indicator determinations (each indicator was assayed in 3 technical replicates). At the mature tuber stage, potato tuber samples were collected and immediately frozen in liquid nitrogen within 5 min after detachment from plants to inhibit enzymatic degradation and maintain initial activity. The frozen samples were then stored in a −80 °C ultra-low temperature freezer (DW-86L626, Haier Group, Qingdao, China) until assay, with a maximum storage period of 1 week to avoid activity loss. Homogenization was performed in an ice bath to maintain low temperature, and PPO/PAL assays were completed within 5 min after centrifugation to avoid interference from non-enzymatic substrate oxidation. Additionally, five pots of plants were randomly selected from each treatment for yield evaluation.

### 2.2. Test Method

#### 2.2.1. Determination of Chlorophyll Content

The concentrations of photosynthetic pigments, including chlorophyll a (Chl a), chlorophyll b (Chl b), and total chlorophyll (Chl a + b), were determined using the method described by Weemaes et al. [27]. Specifically, 0.02 g of potato leaves (taken from the middle of the third fully expanded leaf, avoiding the main vein) were accurately weighed (precision ± 0.001 g) and placed in 10 mL of absolute ethanol (analytical grade, Tianjin Bohuatong Chemical Products Sales Center, Tianjin, China). The mesophyll tissue was kept at 4 °C in the dark for 48 h (shaken twice a day during extraction) until it turned completely white. The absorbance values at 663 nm and 645 nm were measured using an ultraviolet-visible spectrophotometer (Thermo Fisher Scientific 201 PC, Waltham, MA, USA). Each variety (line) was tested in triplicate. Chlorophyll content was calculated using the following formula:

(1a) $$\mathrm{Chl}\;a\;=\;(12.7\;\times \;A663\;\mathrm{nm}-2.69\;\times \;A645\;\mathrm{nm})\;\times \;[V/(10^{3}\;\times \;W)]$$

(1b) $$\mathrm{Chl}\;b=(22.9\;\times \;A645\;\mathrm{nm}-4.68\;\times \;A663\;\mathrm{nm})\;\times \;[V/(10^{3}\;\times \;W)]$$

(1c) $$\mathrm{Chl}\;a+b=(20.21\;\times \;A645\;\mathrm{nm}+8.02\;\times \;A663\;\mathrm{nm})\;\times \;[V/(10^{3}\;\times \;W)]$$

Note: Chl a: the content of chlorophyll a in the sample, in mg·g^−1^ fresh weight (FW); Chl b: chlorophyll b content in the sample, measured in mg·g^−1^ fresh weight (FW); Chl a + b: total chlorophyll content in the sample (sum of chlorophyll a and chlorophyll b); A_663 nm_: absorbance value of chlorophyll a extract at wavelength 663 nm; A_645 nm_: absorbance value of chlorophyll b extract at wavelength 645 nm; *V*: total volume of chlorophyll extract (mL); *W*: chlorophyll leaf fresh weight (g); 10^3^: the unit conversion coefficient, the chlorophyll mass “μg” in the formula is converted to “mg” (1 mg = 1000 μg), so that the final unit is mg·g^−1^.

#### 2.2.2. Browning Index

The browning index was determined according to the method described by Fang et al. [28], with slight modifications. Fresh, mature potato tubers were rinsed with clean water, peeled, and sliced into approximately 0.5 mm thick slices using a slicer (error ± 0.05 mm), avoiding eyes and damaged parts, 3 slices per tuber using a slicer (CL50, Robot Coupe, Vincennes, France). The slices were placed at room temperature (approximately 25 °C). Browning was observed at different time intervals (0 h, 3 h, 5 h, 7 h, 10 h, where 0 h was measured immediately after slicing, and recorded every 1 h thereafter) to assess color change and photographs were taken.

The L*, a*, and b* values of the color difference in the fresh-cut potato slices were measured using a colorimeter (D25NC, Hunter Lab, Reston, VA, USA) to determine the degree of enzymatic browning. A standard whiteboard (L* = 97.06, a* = 0.04, b* = 2.01) was used for calibration and comparison before measuring color difference. Here, L* represents the brightness value, indicating color depth. A higher L* value corresponds to a whiter color, while a lower value indicates a darker color. The a* value reflects the red-green direction of the color: a higher a* value indicates a redder color, and a lower value indicates a greener color. The b* value represents the yellow-blue direction of the color: a higher b* value indicates a yellower color. For each fresh-cut potato slice, three points were randomly selected (upper, middle, and lower sections) for measurement. Each treatment was repeated with three slices, and the L*, a*, and b* values were recorded. The browning index (BI) of the fresh-cut potato slices was calculated, with a higher BI indicating a greater degree of browning.

(2) $$\mathrm{Browning}\;\mathrm{index}=\frac{100\;\times \;(\frac{a*\;+\;1.75\;\times \;L*}{5.645\;\times \;L*\;+\;a*\;-\;3.012\;\times \;b*}-0.31)}{0.172}$$

In this formula: a*: red-green value. Positive values are red, negative values are green; L*: brightness value, from 0 (black) to 100 (white); b*: yellow-blue value. Positive values are yellow, and negative values are blue.

#### 2.2.3. PPO Activity

PPO activity was determined following the method of Zhu et al. [3] with slight modifications. Briefly, 1.0 g of potato tuber samples was weighed, and 2 mL of pre-cooled 0.1 M sodium phosphate buffer (pH 6.5 prepared with analytical grade Na_2_HPO_4_ and NaH_2_PO_4_, Sinopharm Chemical Reagent Co., Ltd., Beijing, China) was added. The mixture was homogenized and filtered. The filtrate was centrifuged at 10,000 rpm for 15 min at 4 °C using a refrigerated centrifuge (Eppendorf 5424 R, Eppendorf AG, Hamburg, Germany). Subsequently, 1.8 mL of buffer, 1 mL of 0.1 mol/L catechol (analytical grade, Shanghai Yuanye Bio-Technology Co., Ltd., Shanghai, China) (freshly prepared, stored in the dark at 4 °C), and 0.2 mL of the supernatant were quickly mixed. A blank control group was set up by replacing the supernatant with an equal volume of buffer. The absorbance was measured at 410 nm using a ultraviolet-visible spectrophotometer (Thermo Fisher Scientific 201 PC, USA). The enzyme solution was stable within 2 h at 4 °C (activity decrease < 5%), and all samples were measured within 30 min after extraction. One unit of enzyme activity (U⋅g^−1^⋅min^−1^) was defined as a change in absorbance of 0.01 per minute.

(3) $$\mathrm{PPO}\;(U\cdot g^{-1}\cdot \min^{-1})=\frac{\Delta 410\;\times {\;V}_{T}}{0.01\;\times \;F_{W}\;\times \;V_{S}\;\times \;t}$$

In the formula: PPO (U⋅g^−1^⋅min^−1^): polyphenol oxidase (PPO) activity in potato tuber samples in units of U⋅g^−1^⋅min^−1^. Δ410: change in absorbance at 410 nm in enzyme-catalyzed reactions. *V_T_*: total supernatant volume (mL). 0.01: absorbance change, PPO enzyme active unit (U). *F_W_*: fresh weight (g) of the potato tuber sample used to extract PPO. *V_S_*: volume of supernatant used (mL). *t*: reaction time for absorbance changes (min).

#### 2.2.4. Total Phenol Content

The total phenol content was determined according to the method of Muñoz-Pina et al. [29] with minor modifications. Briefly, 1.0 g of potato tubers was weighed, and 0.05 g of PVPP (polyvinylpolypyrrolidone, analytical grade, Shanghai Macklin Biochemical Co., Ltd., Shanghai, China) and 3.0 mL of 70% (*v*/*v*) acetone (analytical grade, Tianjin Bohuatong Chemical Products Sales Center, Tianjin, China) were added. The mixture was thoroughly homogenized and extracted at room temperature for 3 h. After centrifugation at 10,000 rpm for 20 min at 4 °C, 0.4 mL of the supernatant, 0.5 mL of Folin–Ciocalteu (diluted 1:10, *v*/*v*) (analytical grade, Beijing Solarbio Science & Technology Co., Ltd., Beijing, China) reagent, and 0.5 mL of 10% (*w*/*v*) Na_2_CO_3_ solution (freshly prepared) (analytical grade, Sinopharm Chemical Reagent Co., Ltd., Beijing, China) were mixed. The solution was diluted to 10 mL with distilled water and incubated in a water bath at 25 °C for 2 h. Absorbance was measured at 765 nm using a ultraviolet-visible spectrophotometer (Thermo Fisher Scientific 201 PC, MA, USA). A standard curve was established with gallic acid (0–1.0 mg/mL, R^2^ = 0.991). The total phenol concentration (C_1_) was determined using a standard curve, and the total phenol content was calculated accordingly.

(4) $$\mathrm{TP}\;(\mathrm{mg}/g)=\frac{C_{1}\;\times \;D\;\times \;V_{T}}{V_{S}\;\times \;F_{W}}$$

In the formula: TP (mg/g): the total phenolic content in the sample in mg/g. C_1_: the concentration of total phenols in the measured solution (mg/mL), obtained from the standard curve. *D*: dilution factor. *V_T_*: total volume of extract (mL). Vs: measure the volume of the supernatant in mL. Fw: fresh weight of the sample (g).

#### 2.2.5. Starch Content

Starch content was determined following the method of Zhang et al. [30] with slight modifications. Briefly, 0.5 g of potato tuber sample was weighed, and 2 mL of deionized water was added. After homogenization, 2 mL of 60% (*v*/*v*) perchloric acid (ultrapure grade, Qingdao Kelong Chemicals Co., Ltd., Qingdao, China) was added, and the mixture was vortexed for 5 min and allowed to stand for 5 min. The volume was then adjusted to 10 mL with distilled water, and the mixture was mixed again for 5 min. After centrifugation at 3700 rpm for 10 min at 4 °C, 50 μL of the supernatant was transferred to a new tube, followed by the addition of 3 mL of deionized water and 1 mL of iodine reagent (0.1 mol/L I_2_ in 0.2 mol/L KI, prepared with analytical grade I_2_ and KI, Tianjin Guangfu Technology Development Co., Ltd., Tianjin, China). The solution was mixed and incubated for 5 min, then diluted to 10 mL and mixed thoroughly. A 200 μL aliquot of the reaction solution was transferred to a microplate, and the optical density (OD) was measured at 660 nm using a multifunctional microplate reader (Synergy HTX, BioTek Instruments, Winooski, VT, USA). Starch content was calculated based on the standard curve.

(5) $$\mathrm{starch}\;\mathrm{content}\;(\%)=\frac{\mathrm{sample}\;\mathrm{concentration}}{\mathrm{Sample}\;\mathrm{quality}\;\times \;\frac{1}{10}\;\times \;\frac{0.5}{10}\;\times {\;10}^{6}}\times \;100$$

#### 2.2.6. Sucrose Content

Sucrose content was determined according to the method of Zhang et al. [31] with minor modifications. Briefly, 0.5 g of potato tuber samples was weighed and homogenized with 1 mL of deionized water (UPT-I-20T, Sichuan Youpu Ultra-Pure Technology Co., Ltd., Chengdu, China). After cooling, the mixture was centrifuged at 15,000 rpm for 10 min at 4 °C using a refrigerated centrifuge (Eppendorf 5424 R, Eppendorf AG, Hamburg, Germany). Subsequently, 50 μL of the supernatant was transferred to a 96-well PCR plate, followed by the addition of 5 μL of 2 g/L NaOH solution (analytical grade NaOH, Sinopharm Chemical Reagent Co., Ltd., Beijing, China). The mixture was thoroughly mixed and incubated at 90 °C for 10 min in a thermal cycler (HWS-24, Shanghai Yiheng Scientific Instruments Co., Ltd., Shanghai, China). Then, 50 μL of 1 g/L resorcinol (analytical grade resorcinol, Analytical Grade, Shanghai Yuanye Bio-Technology Co., Ltd., Shanghai, China) and 150 μL of 10 g/L HCl solution (analytical grade HCl, Sinopharm Chemical Reagent Co., Ltd., Beijing, China) were added, and the solution was incubated in a 90 °C water bath for 10 min. After cooling, the reaction solution was mixed, and 200 μL was transferred to a microplate. The optical density (OD) was measured at 500 nm using a multifunctional microplate reader (Synergy HTX, BioTek Instruments, Winooski, VT, USA). Sucrose content was calculated based on the standard curve (HPLC grade, purity ≥ 99.5%, Shanghai Yuanye Bio-Technology Co., Ltd., Shanghai, China).

(6) $$\mathrm{Sucrose}\;\mathrm{content}\;\left(\mu g/g\right)=\frac{\mathrm{sample}\;\mathrm{concentration}\;\times \;(2\;÷\;\mathrm{sample}\;\mathrm{volume})}{\mathrm{sample}\;\mathrm{mass}\;\times \;10^{6}}$$

#### 2.2.7. Soluble Sugar Content

Soluble sugar content was determined following the method of He et al. [32] with minor modifications. Briefly, 0.5 g of potato tuber samples was weighed and homogenized with 1 mL of deionized water (UPT-I-20T, Sichuan Youpu Ultra-Pure Technology Co., Ltd., Chengdu, China). After cooling, the mixture was centrifuged at 15,000 rpm for 10 min at 4 °C using a refrigerated centrifuge (Eppendorf 5424 R, Eppendorf AG, Hamburg, Germany). A total of 30 μL of the supernatant was transferred to a 96-well PCR plate, followed by the addition of 150 μL of reaction solution C (anthrone-sulfuric acid reagent: prepared by dissolving 0.2 g of anthrone (analytical grade, Shanghai Yuanye Bio-Technology Co., Ltd., Shanghai, China) in 100 mL of concentrated sulfuric acid (98%, analytical grade, Sinopharm Chemical Reagent Co., Ltd., Beijing, China)). After thorough mixing, the plate was incubated in a water bath at 90 °C for 1 min in a thermal cycler (HWS-24, Shanghai Yiheng Scientific Instruments Co., Ltd., Shanghai, China). After cooling, the reaction solution was mixed again, and 200 μL was transferred to a microplate. The optical density (OD) was measured at 630 nm using a multifunctional microplate reader (Synergy HTX, BioTek Instruments, Winooski, VT, USA). Soluble sugar content was calculated based on the standard curve (HPLC grade, purity ≥ 99.5%, Shanghai Yuanye Bio-Technology Co., Ltd., Shanghai, China) (commonly used as a reference for total soluble sugar quantification).

(7) $$\mathrm{Soluble}\;\mathrm{sugar}\;\mathrm{content}\;\left(\mu g/g\right)=\frac{\mathrm{sample}\;\mathrm{concentration}\;\times \;(2\;÷\;\mathrm{sample}\;\mathrm{volume})}{\mathrm{sample}\;\mathrm{mass}\;\times {\;10}^{6}}$$

#### 2.2.8. POD Activity

POD activity was determined according to the method of Pan et al. [33] with minor modifications. Briefly, 0.50 g of potato tubers and 0.06 g of PVPP (PVPP, analytical grade, Shanghai Macklin Biochemical Co., Ltd., Shanghai, China) were homogenized on ice with 2.5 mL of pre-cooled phosphate buffer (0.1 mol/L, pH 6.8, prepared with analytical grade Na_2_HPO_4_ and NaH_2_PO_4_, Sinopharm Chemical Reagent Co., Ltd., Beijing, China). The mixture was then centrifuged at 10,000 rpm for 15 min at 4 °C using a refrigerated centrifuge (Eppendorf 5424 R, Eppendorf AG, Hamburg, Germany). To the supernatant, 2.5 mL of reaction solution (containing 14 μL of guaiacol (analytical grade, Shanghai Yuanye Bio-Technology Co., Ltd., Shanghai, China), 0.5 mL of buffer solution, and 0.5 mL of supernatant were mixed. A blank control group was set up by replacing the supernatant with an equal volume of buffer. The absorbance was immediately measured at 470 nm using a UV–Vis spectrophotometer (Thermo Fisher Scientific 201 PC, Waltham, MA, USA). The reaction system contained 14 μL of guaiacol (analytical grade, Shanghai Yuanye Bio-Technology Co., Ltd., Shanghai, China) and 9.5 μL of 30% (*v*/*v*) H_2_O_2_ (analytical grade, Sinopharm Chemical Reagent Co., Ltd., Beijing, China) dissolved in 25 mL of pH 6.0 phosphate buffer). One unit of enzyme activity (U) was defined as an increase of 0.01 in Δ470 per minute, expressed as U^−1^g^−1^min.

(8) $$\mathrm{POD}\;(U^{-1}g^{-1}\min)=\frac{\Delta 470\;\times \;V_{T}}{0.01\;\times \;F_{W}\;\times {\;V}_{S}\;\times \;t}$$

In the formula: Δ470 signifies the absorbance at 470 nm; *V_T_* represents the extract’s total volume; *F_W_* stands for the weight of the sample; *V_S_* denotes the volume of enzyme solution utilized in the measurement process; *t* is the reaction time (min); every 0.01 increment in Δ470 equates to a single unit of enzyme activity (U).

#### 2.2.9. PAL Activity

PAL activity was determined following the method of Li et al. [34] with minor modifications. Briefly, 0.5 g of potato tubers and 0.06 g of PVPP (analytical grade, Shanghai Macklin Biochemical Co., Ltd., Shanghai, China) were placed in a 15 mL centrifuge tube, and 2 mL of pre-cooled boric acid buffer (pH 8.5, prepared with analytical grade boric acid and sodium borate, Sinopharm Chemical Reagent Co., Ltd., Beijing, China) containing 0.4 μL/mL mercaptoethanol (analytical grade, Shanghai Yuanye Bio-Technology Co., Ltd., Shanghai, China) was added. The mixture was homogenized on ice and centrifuged at 10,000 rpm for 15 min at 4 °C using a refrigerated centrifuge (Eppendorf 5424 R, Eppendorf AG, Hamburg, Germany). Then, 3 mL of boric acid buffer, 3 mL of 0.02 mol/L L-phenylalanine (HPLC grade, purity ≥ 98%, Shanghai Yuanye Bio-Technology Co., Ltd., Shanghai, China), and 0.5 mL of the supernatant were mixed. A blank control group was set up by replacing the supernatant with an equal volume of buffer. The reaction was carried out in a water bath at 40 °C for 1 h, and the reaction was terminated by the addition of 0.1 mol/L hydrochloric acid (analytical grade, Sinopharm Chemical Reagent Co., Ltd., Beijing, China). The absorbance before and after the incubation was measured at 290 nm using a UV–Vis spectrophotometer (Thermo Fisher Scientific 201 PC, Waltham, MA, USA). One unit of enzyme activity (U) was defined as an increase in absorbance of 0.01 per hour, and the results were expressed as U^−1^g^−1^min.

(9) $${\mathrm{PAL}\;(U}^{-1}g^{-1}\min)=\frac{\Delta 290\;\times \;V_{T}}{0.01\;\times \;F_{W}\;\times \;V_{S}\;\times \;t}$$

The formula includes: Δ290 for absorbance at 290 nm; *V_T_* for the extract’s total volume (mL); *F_W_* for the sample’s fresh weight (g); *V_S_* for the enzyme solution’s volume utilized in the analysis (mL); *t* for reaction time (min).

#### 2.2.10. MDA Content

MDA content was determined according to the method described by Kong et al. [35]. Briefly, 0.8 g of potato tubers was ground in 4 mL of 100 g/L trichloroacetic acid solution (TCA, analytical grade, Sinopharm Chemical Reagent Co., Ltd., Beijing, China) and centrifuged at 10,000 rpm for 20 min at 4 °C using a refrigerated centrifuge (Eppendorf 5424 R, Eppendorf AG, Hamburg, Germany). Then, 2 mL of the supernatant and 2 mL of 0.67% (*w*/*v*) thiobarbituric acid solution (TBA, analytical grade, Shanghai Macklin Biochemical Co., Ltd., Shanghai, China) were thoroughly mixed and boiled for 20 min. After cooling to room temperature, the mixture was centrifuged again at 10,000 rpm for 20 min at 4 °C. The absorbance of the reaction mixture was measured at 450 nm, 532 nm, and 600 nm using a UV–Vis spectrophotometer (Thermo Fisher Scientific 201 PC, Waltham, MA, USA), The MDA content was expressed as μmol/kg.

(10a) C=6.45 × (A−B)−0.56 × D

(10b) $$x=\frac{C\;\times \;V}{V_{S}\;\times \;m\;\times \;10^{3}}$$

The formula includes: C for malondialdehyde concentration; V for total extract volume; *V_S_* for the volume of liquid added to the sample during measurement; m for the weight of the potato sample; X for MDA content; A for absorbance at 532 nm; B for 600 nm absorbance; D for 450 nm.

#### 2.2.11. Organic Acid Content

The content of organic acids was determined according to the method of Ikegaya et al. [36]. A standard solution was prepared by accurately weighing 10 mg of citric acid and ascorbic acid (HPLC grade, purity ≥ 99%, Beijing Solarbio Science & Technology Co., Ltd., Beijing, China), dissolving them in water, and diluting to a final volume of 10 mL in a 10 mL centrifuge tube. A 0.2 g potato sample was ground with 2 mL of ultrapure water, transferred to a 2 mL centrifuge tube, and ultrasonically extracted (200 W, ice bath) for 30 min with intermittent shaking. The mixture was then centrifuged at 12,000 rpm for 5 min at 4 °C. The supernatant was filtered through a 0.22 μm filter membrane and analyzed by high-performance liquid chromatography (Agilent 1260 II, Agilent Technologies, Santa Clara, CA, USA). Mobile phase A: 10% methanol; mobile phase B: 90% phosphoric acid. Chromatographic column: XSelect HSS T3 column (4.6 mm × 250 mm, 5 μm; Waters Corporation, Milford, MA, USA). Injection volume: 5 μL. Column temperature: 30 °C. Detection wavelengths: citric acid 210 nm, ascorbic acid 245 nm. Flow rate: 1 mL/min.

#### 2.2.12. Chlorogenic Acid Content

Chlorogenic acid content in potato tubers was determined following the method of Zhou et al. [37] with slight modifications. A 10 mg standard of chlorogenic acid (HPLC grade, purity ≥ 98%, Beijing Solarbio Science & Technology Co., Ltd., Beijing, China) was accurately weighed, dissolved in methanol, and diluted to 10 mL to prepare a 1 mg/mL standard solution. A standard curve was established with R^2^ = 0.9998. A 0.2 g potato sample was ground in liquid nitrogen, and 2 mL of chromatographic methanol (HPLC grade, Tianjin Bohuatong Chemical Products Sales Center, Tianjin, China) was added. The mixture was ultrasonically extracted (200 W, ice bath) for 1 h with intermittent vortexing, then centrifuged at 12,000 rpm for 5 min at 4 °C using a refrigerated centrifuge (Eppendorf 5424 R, Eppendorf AG, Hamburg, Germany). The supernatant was filtered through a 0.22 μm microporous membrane and analyzed by high-performance liquid chromatography (Agilent 1260 II, Agilent Technologies, Santa Clara, CA, USA). Mobile phase A: 68% acetonitrile; mobile phase B: 32% of 0.1% phosphoric acid. Chromatographic column: Symmetry C18 column (4.6 mm × 250 mm, 5 μm; Waters Corporation, Milford, MA, USA) Injection volume: 5 μL. Column temperature: 30 °C. Detection wavelength: chlorogenic acid: 292 nm. Flow rate: 1 mL/min.

#### 2.2.13. Amino Acid Content

Free amino acids were extracted and analyzed by high-performance liquid chromatography according to the method reported by Dong et al. [38]. A 0.2 g potato sample was ground in liquid nitrogen and mixed with 1 mL of 0.5 mol/L hydrochloric acid (analytical grade, Sinopharm Chemical Reagent Co., Ltd., Beijing, China) (pre-cooled to 4 °C). The sample was ultrasonically treated for 20 min (200 W, ice bath), shaking every 5 min. After centrifugation at 12,000 rpm for 20 min at 4 °C, the supernatant was collected. The supernatant was diluted 10 times, filtered through a 0.22 μm aqueous-phase filter membrane, and analyzed using an automatic amino acid analyzer (Chromaster 5510, Hitachi High-Tech Corporation, Tokyo, Japan; column temperature: 50 °C; detection wavelength: 570 nm for most amino acids, 440 nm for proline; mobile phase flow rate: 0.1 mL/min; pre-column derivatization: ninhydrin, reaction temperature: 50 °C, reaction time: 30 min).

#### 2.2.14. Statistical Analysis

Data organization was conducted using Microsoft Office 2016. The least significant difference (LSD) test and Duncan’s multiple range test at the 0.05 significance level were performed using SPSS statistical software version 20 (IBM Corporation, Armonk, NY, USA) for post hoc comparison of treatment means, and different lowercase letters were used in the table/graph to indicate significant differences. Data visualization and plotting were carried out using OriginPro 2024 (OriginLab Corporation, Northampton, MA, USA).

## 3. Results and Analysis

### 3.1. Effects of Copper on the Contents of Starch, Sucrose, and Soluble Sugar in Tubers

**Figure 1:**
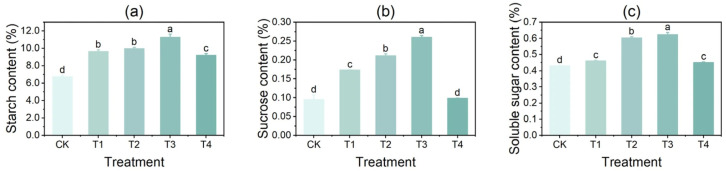
Effects of different concentrations of CuSO_4_ on the contents of starch, sucrose, and soluble sugar in potato tubers. (**a**) Starch content; (**b**) sucrose content; (**c**) soluble sugar content. Significant differences (*p* < 0.05) are indicated by lowercase letters, starting from the highest value in alphabetical order. Identical letters indicate no significant difference. All error bars represent the mean ± standard deviation (n = 3).

Following the T1, T2, and T3 treatments, the contents of starch, sucrose, and soluble sugar in potato tubers exhibited an increasing trend (Figure 1). The highest levels of starch, sucrose, and soluble sugar were observed under the T3 treatment, with values of 11.31%, 0.26%, and 0.62%, respectively. These were significantly higher by 66.7%, 122.11%, and 44.55%, respectively, compared to the control (CK). However, under the high-concentration T4 treatment, the accumulation of starch, sucrose, and soluble sugar in the tubers was inhibited, with reductions of 18.25%, 61.92%, and 27.77%, respectively, compared to T3. The starch and soluble sugar contents in the tubers under T4 treatment were significantly higher by 36.3% and 4.41%, respectively, compared to CK, and the sucrose content did not change significantly.

### 3.2. Effect of Copper on the Content of Citric Acid, Ascorbic Acid, and Chlorogenic Acid in Tubers

**Figure 2:**
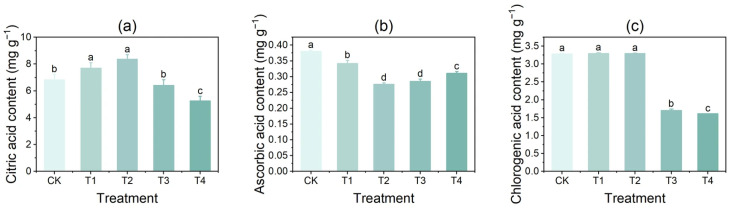
Effects of different CuSO_4_ concentrations on the contents of citric acid, ascorbic acid, and chlorogenic acid in potato tubers. (**a**) citric acid; (**b**) ascorbic acid; (**c**) chlorogenic acid. Significant differences (*p* < 0.05) are indicated by lowercase letters in alphabetical order, starting from the highest value. Identical letters denote no significant difference. Error bars represent the mean ± standard deviation (SD) of three replicates (n = 3).

With increasing CuSO_4_ concentration, the citric acid content in potato tubers initially increased and then declined, whereas the ascorbic acid content first decreased and subsequently increased. Compared with CK, the citric acid content increased significantly by 12.93% and 22.65% under the T1 and T2 treatments, respectively. However, following the T3 and T4 treatments, the citric acid content decreased significantly by 5.97% and 22.99%, respectively (Figure 2a). In contrast, ascorbic acid content in the tubers was significantly reduced under all treatments, showing decreases of 10.18%, 27.37%, 24.91%, and 18.33% for T1, T2, T3, and T4, respectively, compared with CK (Figure 2b). For chlorogenic acid, no significant difference was observed between T1 or T2 and CK; however, its content decreased markedly by 48.21% and 50.96% under the T3 and T4 treatments, respectively, compared with CK (Figure 2c).

### 3.3. Effect of Copper on the Content of Free Amino Acids in Tubers

**Figure 3:**
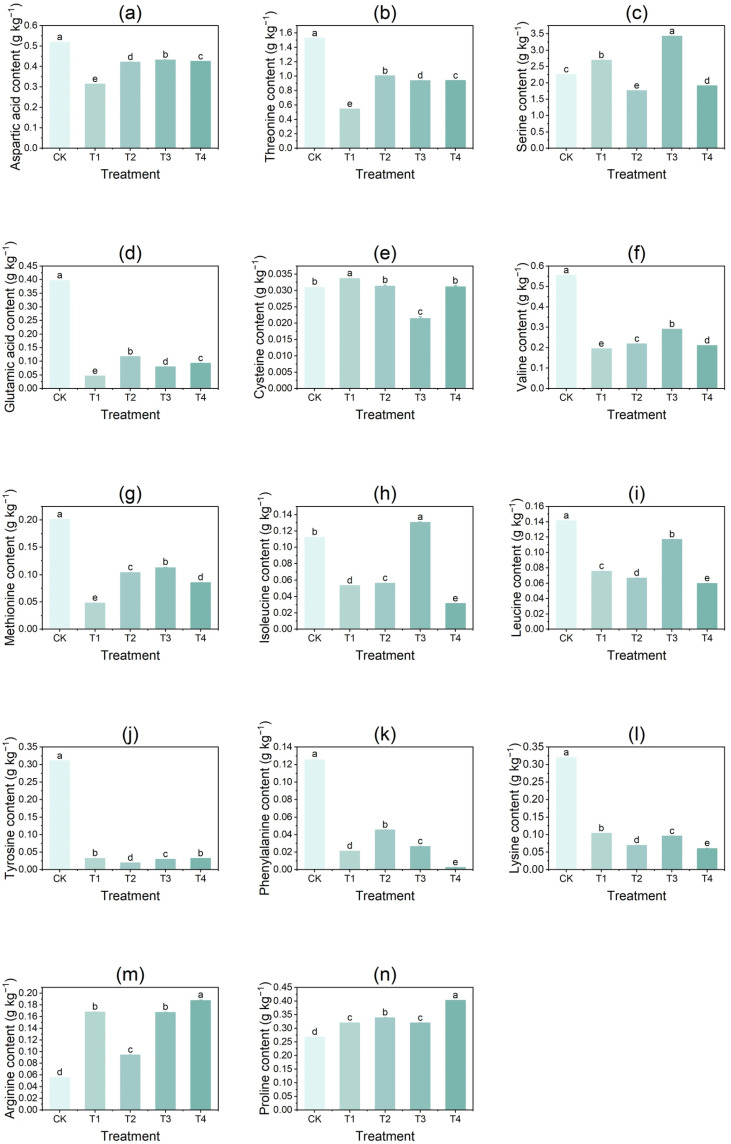
Effects of different concentrations of CuSO_4_ treatment on free amino acid content in potato tubers. (**a**) aspartic acid; (**b**) threonine; (**c**) serine; (**d**) glutamic acid; (**e**) cysteine; (**f**) valine; (**g**) methionine; (**h**) isoleucine; (**i**) leucine; (**j**) tyrosine; (**k**) phenylalanine; (**l**) lysine; (**m**) arginine; (**n**) proline. Data are presented as the mean ± standard error (SE) of three replicates. Different letters indicate significant differences at *p* < 0.05.

With increasing CuSO_4_ concentration, the contents of aspartic acid, threonine, glutamic acid, valine, methionine, leucine, tyrosine, phenylalanine, and lysine in potato tubers decreased significantly, whereas the contents of arginine and proline increased significantly (Figure 3). The levels of serine, cysteine, and isoleucine exhibited an initial increase followed by a decline. Compared with CK, aspartic acid, threonine, glutamic acid, valine, and methionine contents were significantly reduced under all treatments (T1–T4), with the greatest decreases observed under T1 treatment (39.5%, 64.4%, 88.5%, 64.9%, and 76.1%, respectively). Leucine, phenylalanine, and lysine contents were also significantly lower than CK across treatments, with the largest reductions occurring under T4 (57.7%, 98.1%, and 81.3%, respectively). Tyrosine content decreased significantly under all treatments, with the largest reduction (93.9%) under T2. In contrast, arginine and proline contents were significantly higher than CK under T1–T4, with the greatest increases under T4 (238.46% and 50.36%, respectively). For serine, the highest content was observed under T3 (3.246 μg/mg), representing a 52.0% increase compared with CK, while the lowest was under T2 (1.759 μg/mg), a 22.0% reduction. The highest cysteine content (0.034 μg/mg) occurred under T1, whereas the lowest (0.021 μg/mg) was detected under T3. Similarly, isoleucine content peaked under T3 (0.130 μg/mg) but was lowest under T4 (0.031 μg/mg).

### 3.4. Effects of Copper on Potato Growth and Yield

With increasing CuSO_4_ concentration, potato plant height, stem diameter, tuber number per plant, yield per plant, and total chlorophyll content initially increased and then declined. Under CK treatment, all five parameters were the lowest (Table 2). Compared with CK, T2 treatment resulted in the highest plant height, stem diameter, yield per plant, and total chlorophyll content, which increased significantly by 9.92%, 80.45%, 117.01%, and 3.99%, respectively. The highest tuber number per plant was observed under the T3 treatment, showing a significant increase of 78.57% compared with CK.

### 3.5. Effect of Copper on PPO Activity and Total Phenol Content in Tubers

**Figure 4:**
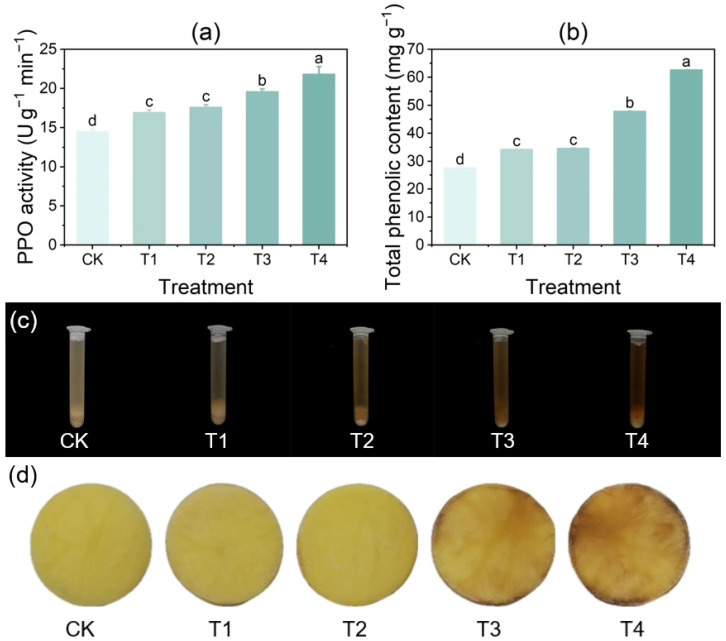
Effects of different concentrations of CuSO_4_ on PPO activity and total phenol content in potato tubers. (**a**) PPO activity; (**b**) total phenol content; (**c**) tuber slurry incubated at room temperature for 10 h; (**d**) tuber slices observed at room temperature for 10 h. Data are presented as mean ± standard error (SE) of three replicates. Different letters indicate significant differences at *p* < 0.05.

The PPO activity and total phenol content of potato tubers increased with rising CuSO_4_ concentration (Figure 4). Under CK treatment, both PPO activity and total phenol content were the lowest. In contrast, under T4 treatment, PPO activity and total phenol content reached the highest levels, showing significant increases of 50.77% and 127.1%, respectively, compared with CK. PPO activity under the T1, T2, and T3 treatments increased significantly by 16.92%, 21.54%, and 35.38%, respectively, relative to CK. Similarly, the total phenol content increased significantly under the T1, T2, and T3 treatments by 23.99%, 25.28%, and 73.31%, respectively, compared with CK.

### 3.6. Effects of Copper on Membrane Lipid Peroxidation and Browning-Related Enzyme Activities in Potato Tubers

**Figure 5:**
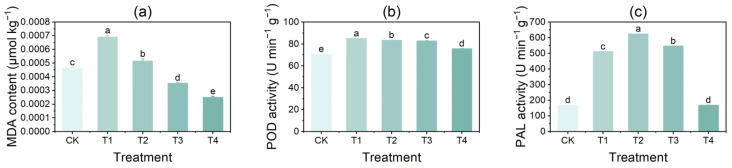
Effects of different concentrations of CuSO_4_ on membrane lipid peroxidation and browning-related enzyme activities in potato tubers. (**a**) Malondialdehyde (MDA) content; (**b**) peroxidase (POD) activity; (**c**) phenylalanine ammonia-lyase (PAL) activity. Data are expressed as mean ± standard error (SE) of three replicates. Different letters indicate significant differences at *p* < 0.05.

With increasing CuSO_4_ concentration, MDA content as well as POD and PAL activities in potato tubers first increased and then declined. Following T1 treatment, MDA content and POD activity reached their maximum levels, showing significant increases of 49.98% and 20.85%, respectively, compared with CK (Figure 5a,b). Under T2 treatment, PAL activity was the highest (623.47 μg/g), which represented a significant 269.94% increase compared with CK (Figure 5c).

### 3.7. Effects of Copper on Color Parameters and Browning Index of Potato Tubers

With increasing CuSO_4_ concentration, the L* value of potato tubers decreased, while the a*, b*, and BI (Browning index) values increased (Table 3). The L* value was highest under CK treatment, whereas under T4 treatment it was the lowest, showing a significant reduction of 8.38% compared with CK. The a*, b*, and BI values were lowest in CK, but under T2 treatment, they were significantly increased by 62.01%, 6.16%, and 18.13%, respectively, relative to CK. The highest a*, b*, and BI values were observed under T4 treatment, with significant increases of 112.36%, 49.13%, and 80.87%, respectively, compared with CK.

### 3.8. Correlation Analysis

**Figure 6:**
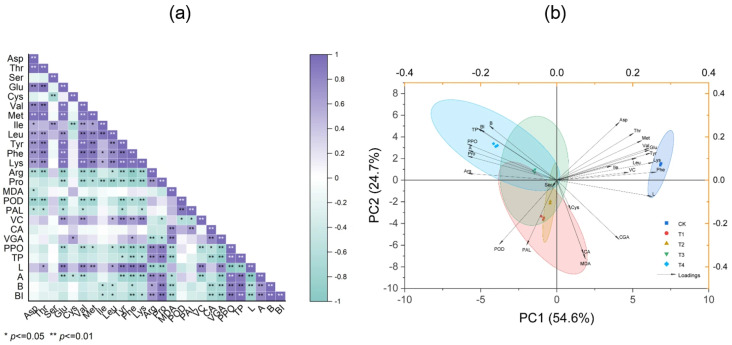
Correlation analysis and principal component analysis. (**a**) The heatmap shows the Pearson correlation analysis of the observed parameters under CuSO_4_ treatment; (**b**) the score plots and loading plots represent the PCA results of the observed parameters after CuSO_4_ treatment. Variables: Asp, Aspartic acid; Thr, Threonine; Ser, Serine; Glu, Glutamic acid; Cys, Cysteine; Val, Valine; Met, Methionine; Ile, Isoleucine; Leu, Leucine; Tyr, Tyrosine; Phe, Phenylalanine; Lys, Lysine; Arg, Arginine; Pro, Proline; MDA, Malondialdehyde; POD, Peroxidase; PAL, Phenylalanine ammonia-lyase; VC, Ascorbic acid; CA, Citric acid; CGA, Chlorogenic acid; PPO, Polyphenol oxidase; TP, Total phenol; BI, Browning index; L, L value; a, A value; b, B value. * and ** indicate that the correlation coefficients were significant at the *p* < 0.05 and 0.01 levels, respectively.

Correlation analysis indicated that the browning index (BI) was synergistically regulated by secondary metabolic enzyme activities, organic acid accumulation, and amino acid metabolism (Figure 6a). Among the secondary metabolic enzymes, PPO activity was significantly and positively correlated with BI. In terms of organic acids, citric acid and chlorogenic acid contents were significantly and negatively correlated with BI. Within amino acid metabolism, phenylalanine (Phe), isoleucine, leucine, and lysine were significantly negatively correlated with BI, whereas arginine and proline showed significant positive correlations. Additionally, the total phenol content was significantly positively correlated with BI, while the lipid peroxidation marker malondialdehyde (MDA) was significantly negatively correlated. Furthermore, PPO activity was significantly positively correlated with arginine and proline contents, and significantly negatively correlated with glutamic acid, valine, tyrosine, phenylalanine, lysine, MDA, ascorbic acid, and chlorogenic acid contents.

The first two principal components accounted for 79.3% of the total variance (Figure 6b). The distribution of amino acids, organic acids, and enzyme activity variables was relatively dispersed, while TP, PPO, Pro, and Arg exhibited strong positive associations with the color parameters (A and B) and the browning index (BI). There were significant differences in metabolic phenotypes among different treatments (T1–T4).

### 3.9. Correlation Analysis of Potato Tuber Browning Index and Key Metabolite Activity

**Figure 7:**
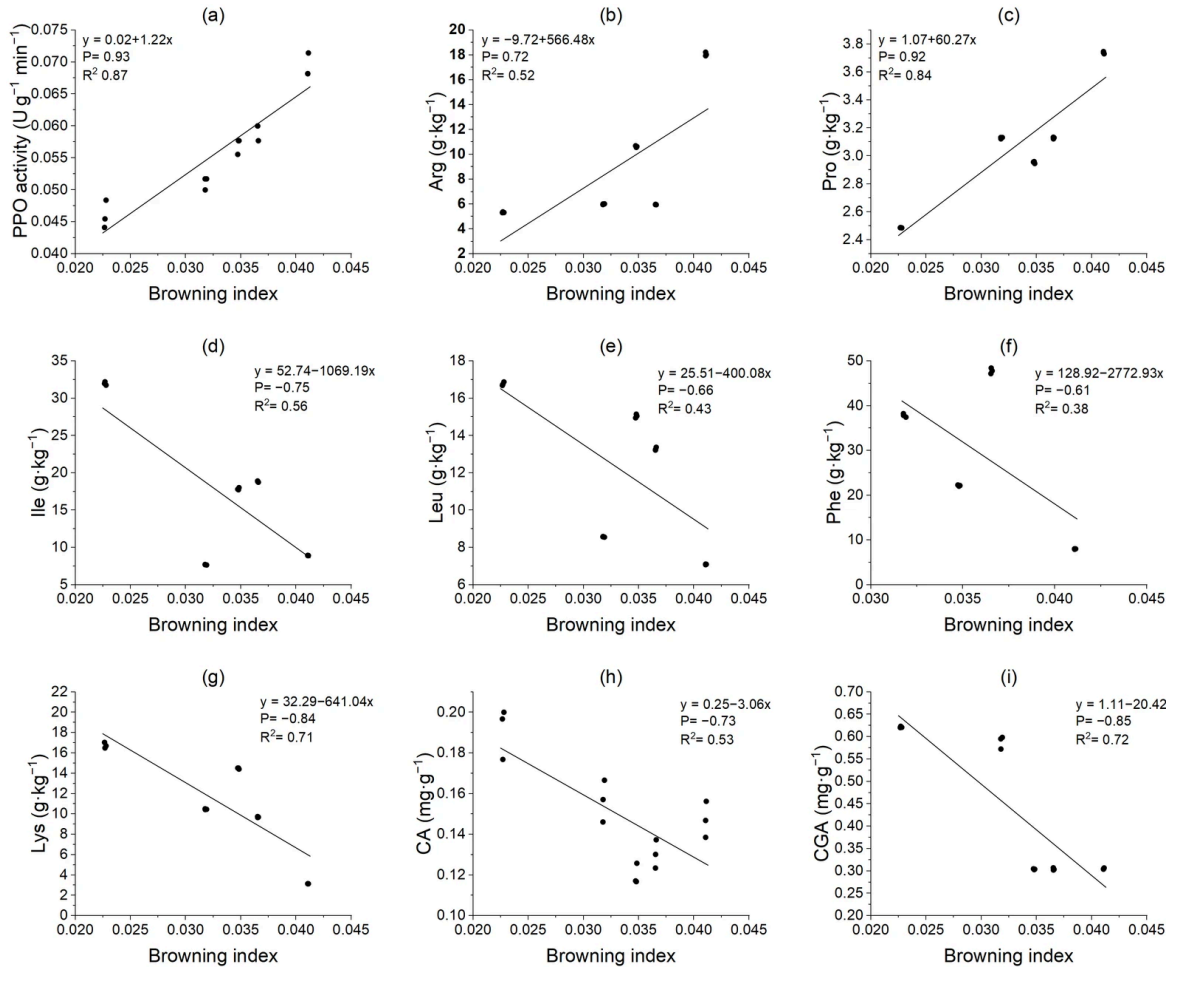
Linear fitting relationships between polyphenol oxidase activity (**a**), free amino acids (**b**–**g**), organic acids (**h**,**i**) and the browning index (BI) of potato tubers.

As shown in Figure 7, the browning index (BI) of potato tubers was significantly correlated with PPO activity, key free amino acids, and organic acid contents. The PPO activity in potato tubers (R^2^ = 0.87), Arg content (R^2^ = 0.52), and Pro content (R^2^ = 0.84) were significantly positively correlated with the browning index. Conversely, the contents of Ile, Leu, Phe, and Lys in the tubers (R^2^ = 0.38~0.71) were negatively correlated with the BI; the contents of organic acids CA and CGA were negatively correlated with the BI at an extremely significant level (R^2^ = 0.53, 0.72).

### 3.10. Copper-Mediated Metabolic Pathways Driving Enzymatic Browning in Potato Tubers

**Figure 8:**
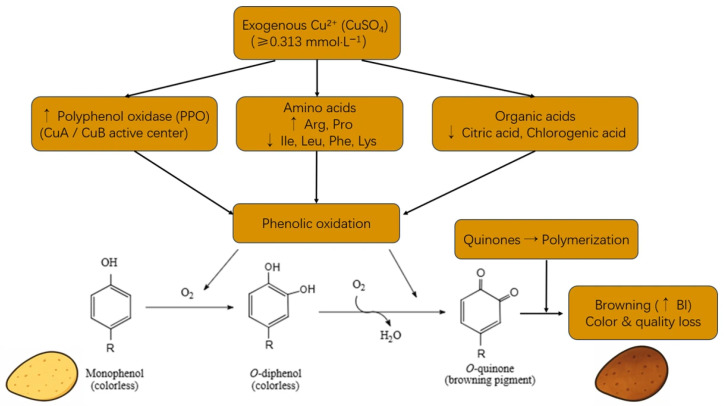
Mechanism of Cu^2+^-induced enzymatic browning in potato tubers. Cu^2+^ enhances polyphenol oxidase (PPO) activity, promoting the oxidation of phenolics. It also modulates metabolism: arginine (Arg) and proline (Pro) increased, showing positive correlations with the browning index (BI), whereas isoleucine (Ile), leucine (Leu), phenylalanine (Phe), lysine (Lys), citric acid, and chlorogenic acid decreased, showing negative correlations with BI. These metabolic changes accelerate quinone polymerization into melanin, leading to increased BI and reduced tuber quality, particularly at CuSO_4_ concentrations ≥ 0.313 mmol·L^−1^.

Exogenous copper sulfate (Cu^2+^) regulates the enzymatic browning of potato tubers through multiple metabolic pathways (Figure 8). Copper ions directly interact with the active center of polyphenol oxidase (PPO), enhancing its catalytic activity, and influence browning by modulating amino acid and organic acid metabolism. These metabolic changes collectively promote phenolic oxidation and quinone polymerization, ultimately leading to melanin formation, manifested as tuber browning, color deterioration, and reduced quality. In the present study, when copper ion concentrations exceeded 0.313 mmol·L^−1^, these effects were particularly pronounced, indicating a concentration-dependent regulatory role of copper in enzymatic browning.

## 4. Discussion

### 4.1. Effects of Copper Ions on the Growth and Development of Potato Plants

Copper is an essential micronutrient for plants and plays a critical role in the growth and development of potato [39]. An appropriate supply of copper helps maintain the stability of chloroplast ultrastructure, thereby enhancing photosynthetic efficiency and promoting biomass accumulation [40]. However, excessive copper induces the overproduction of reactive oxygen species (ROS) in cells, leading to membrane lipid peroxidation, disruption of cell membrane integrity, and ultimately inhibiting normal plant growth [41].

In the present study, application of 0.235 mmol·L^−1^ exogenous copper significantly increased the chlorophyll content in potato leaves, promoted shoot growth, enhanced photosynthetic capacity, and accelerated the transport of photosynthetic assimilation products from source leaves to tubers, thereby increasing tuber yield. Conversely, when copper concentrations were below the optimal level, plants exhibited chlorosis of young leaves, stunted growth, and impaired transport of photosynthates to tubers, resulting in a reduced number of tubers per plant. When copper concentrations exceeded 0.235 mmol·L^−1^, potato growth was markedly inhibited. Plants showed reduced height, leaf-edge necrosis, decreased chlorophyll content, and significantly lower tuber yield, likely due to ROS accumulation and oxidative stress induced by high copper levels. In addition, high copper concentrations have been reported to damage roots, reducing lateral root formation, decreasing water and nutrient uptake efficiency, and further exacerbating adverse effects on plant growth and yield [42,43].

### 4.2. Effects of Copper Ions on Potato Tuber Quality

The supply of copper ions exerted multi-dimensional regulatory effects on potato tuber quality, showing a clear concentration-dependent response. As a cofactor of various key enzymes in carbon metabolism, copper can enhance the efficiency of photosynthetic electron transport and respiratory metabolism, thereby promoting the conversion of photosynthetic products into starch while preventing excessive sugar accumulation, ultimately improving the nutritional quality and processing suitability of tubers [44,45]. Starch, as the main carbohydrate, plays a crucial role in regulating the glycemic index. An increase in sucrose content will moderately raise the glycemic index; soluble sugars directly affect the changes in the glycemic index. In the present study, tuber contents of starch, sucrose, and soluble sugar were significantly increased under 0.235 mmol·L^−1^ Cu^2+^ treatment, followed by 0.157 mmol·L^−1^. When copper concentrations exceeded 0.235 mmol·L^−1^, starch, sucrose, and soluble sugar contents in tubers decreased significantly. Appropriate copper levels promote tuber starch accumulation by improving the supply of photosynthates and ATP production efficiency while maintaining reducing sugar levels below the processing threshold (≤0.20 mg·g^−1^), thereby enhancing the processing quality of potatoes [46,47]. However, excessive copper induces the accumulation of reactive oxygen species (ROS), disrupts chloroplast structure, and inhibits the activities of starch synthase and key sugar-metabolizing enzymes. These effects reduce the supply of photosynthetic products and lead to significant declines in starch, sucrose, and soluble sugar contents in tubers. Simultaneously, decreases in dry matter content and deterioration in flavor and taste further limit improvements in tuber yield and quality [48].

### 4.3. Effects of Copper Ions on Potato Tuber Browning-Related Enzyme Activity

As an essential mineral element, copper ions exert a significant concentration-dependent regulation on the activities of browning-related enzymes in potato tubers, directly affecting enzymatic browning [49]. Among these enzymes, polyphenol oxidase (PPO) is the core enzyme catalyzing the browning reaction. PPO is a copper-containing terminal oxidoreductase, with its active site composed of copper ions. Copper is not only essential for maintaining its catalytic function but also modulates its substrate affinity. PPO catalyzes the oxidation of phenolic compounds in tubers to o-quinones, which subsequently polymerize into melanin, leading to browning [50,51]. In the present study, appropriate copper concentrations (0.078–0.157 mmol·L^−1^) significantly increased PPO activity, consistent with previous reports that elevated copper promotes PPO activity. However, excessive copper may induce the accumulation of reactive oxygen species (ROS) or disrupt enzyme conformational stability, causing PPO activity imbalance and further enhancing phenolic oxidation [52,53].

Peroxidase (POD) is widely present in plant tissues and closely associated with respiration, photosynthesis, and oxidative metabolism [54,55]. Although POD is not the dominant enzyme in tuber browning, in the presence of hydrogen peroxide, it catalyzes the oxidation of phenolic compounds to quinones, which subsequently polymerize into pigments [56]. POD also functions as an important antioxidant enzyme, scavenging excess free radicals and delaying membrane lipid peroxidation, thereby protecting the cellular membrane system [57,58]. In this study, POD activity first increased and then decreased with rising copper concentrations, with all treatments from 0.078 to 0.313 mmol·L^−1^ significantly higher than the control. However, in our study, despite elevated POD activity under high Cu^2+^ (0.313 mmol·L^−1^), MDA content (a marker of lipid peroxidation) still increased, indicating that potato POD’s antioxidant capacity is insufficient to counteract ROS accumulation. This may be due to the dominance of browning-related POD isozymes in potato tubers [59]. This suggests that copper may induce stress resistance in tubers and activate the antioxidant defense system within an optimal range, allowing POD to function both as an antioxidant and as a participant in phenol oxidation in the presence of hydrogen peroxide. Excess Cu^2+^ disrupts redox homeostasis, elevating ROS levels and inducing the oxidative modification of PPO, thereby altering its conformation and catalytic efficiency. This shift diverts carbon flux toward phenolic synthesis via enhanced PAL activity, intensifying browning. Conversely, moderate Cu supply maintains ROS at signaling levels that favor antioxidant enzyme activation and balanced carbon allocation [8].

Phenylalanine ammonia lyase (PAL), a rate-limiting enzyme in the phenylpropanoid pathway, catalyzes the formation of phenolic compounds from L-phenylalanine, thereby affecting the availability of substrates for browning [60]. In this study, PAL activity initially increased and then decreased under copper treatment, consistent with the general regulation of Cu^2+^ on plant PAL activity [61]. Appropriate copper concentrations can activate PAL, promote the synthesis of phenolic precursors, and provide substrates for enzymatic browning. Moreover, as a defense-related enzyme, increased PAL activity may indirectly mitigate membrane lipid damage by enhancing ROS scavenging, thereby partially reducing browning in fresh-cut tubers [62].

### 4.4. Regulation of Organic Acids by Copper Ions and Their Effects on Enzymatic Browning

Organic acids are important effector molecules mediating the regulation of enzymatic browning by copper ions. As the essential cofactors of polyphenol oxidase (PPO), copper ions not only directly affect PPO activity but also indirectly modulate the browning process by altering the accumulation of organic acids.

Citric acid is a typical anti-browning organic acid that can delay browning through multiple mechanisms, including scavenging free radicals, chelating Cu^2+^ at the PPO active center, lowering pH, and reducing oxygen solubility, thereby inhibiting enzymatic reactions [63,64]. This study observed that the regulation of copper concentration on the accumulation of citric acid in potato tubers exhibited a significant dose-dependent characteristic: within the appropriate copper concentration range (0–0.157 mmol·L^−1^), it could significantly drive the accumulation of citric acid in the tubers, while when the concentration increased to 0.235–0.313 mmol·L^−1^, the synthesis process was significantly inhibited. This suggests that copper may mitigate PPO activity by elevating citric acid levels, decreasing pH, or chelating the PPO active center, thus slowing browning [65,66]. Ascorbic acid, a strong antioxidant, also plays a key role in regulating enzymatic browning. Its mechanisms include reducing quinone intermediates generated during browning and competitively binding to the PPO active center, thereby preventing further polymerization [67]. Copper ions can reduce the stability of vitamins. This might be because copper ions act as redox catalysts and directly participate in vitamin oxidation reactions, inducing the generation of reactive oxygen species (ROS) and triggering oxidative degradation. At the same time, they form coordination complexes with functional groups such as hydroxyl, amino, and carboxyl groups in the vitamin molecules, altering their spatial conformation and reducing their structural stability, making them more prone to oxidation or hydrolysis [68]. This may inhibit PPO activity through Cu^2+^ chelation at the PPO active center or pH reduction, ultimately reducing browning [69]. Chlorogenic acid, a phenolic substrate, is readily oxidized under PPO catalysis to produce colored products, making it a critical metabolite that promotes browning [70]. Our results showed that low copper concentrations (0–0.157 mmol·L^−1^) significantly enhanced chlorogenic acid accumulation in tubers, whereas high concentrations (0.235–0.313 mmol·L^−1^) suppressed its synthesis. This may be explained by the fact that low copper concentrations facilitate carbon flux toward chlorogenic acid synthesis in the phenylpropanoid pathway, while higher concentrations accelerate its oxidative degradation through ROS accumulation and elevated PPO activity, leading to reduced levels [71]. The increase in organic acid content in potato tubers significantly affects the taste and quality after cooking. As the core substance determining the sourness, it is positively correlated with the intensity of sour taste perception [72]. In summary, copper ions exert a bidirectional regulatory effect on enzymatic browning by modulating the accumulation of organic acids such as citric acid, ascorbic acid, and chlorogenic acid. The direction and magnitude of this effect depend strongly on the copper supply level.

### 4.5. Regulation of Amino Acids by Copper Ions and Their Effects on Enzymatic Browning

Amino acids play an important regulatory role in the enzymatic browning of potato tubers as their accumulation and distribution directly influence browning-related enzyme activity and substrate availability. In this study, the levels of arginine and proline were significantly and positively correlated with the browning index, whereas isoleucine, leucine, phenylalanine, and lysine were significantly and negatively correlated with the browning index.

Specifically, all concentrations of copper ion treatment markedly increased the arginine and proline levels, both of which were positively associated with PPO activity and total phenolic content. The increase of proline may result from Cu-induced activation of Δ^1^-pyrroline-5-carboxylate synthase (P5CS), contributing to osmoprotection and ROS scavenging. Meanwhile, phenylalanine serves as a PAL substrate for phenolic biosynthesis, linking Cu regulation to carbon flux reallocation and browning progression [73]. This suggests that arginine and proline may exacerbate browning by participating in oxidation reactions, enhancing PPO catalytic efficiency, and providing additional phenolic substrates [74]. These findings are largely consistent with previous reports.

Conversely, copper ion treatment significantly reduced the levels of isoleucine, leucine, phenylalanine, and lysine, which were negatively correlated with PPO activity and total phenolic content. These amino acids are generally recognized for their antioxidant properties, as they can inhibit enzymatic browning by chelating Cu^2+^ at the PPO active site or directly scavenging free radicals, thereby reducing phenolic substrate oxidation [14,23,75,76].

### 4.6. Future Research Directions

This study demonstrates that copper ions exert a bidirectional regulatory effect on amino acid metabolism, enhancing enzymatic browning through the accumulation of arginine and proline while the reduction of isoleucine, leucine, phenylalanine, and lysine plays an inhibitory role. This complexity underscores the need to further elucidate the copper-mediated metabolic network underlying browning regulation. Future research should integrate metabolomic, transcriptomic, and enzymological analyses to uncover the molecular mechanisms through which copper regulates amino acid and organic acid metabolism and affects PPO activity.

In addition, the synergistic interactions among amino acids, organic acids, and antioxidants merit systematic investigation to construct a comprehensive model of copper-induced browning regulation. Although this study focused on controlled pot experiments, field-based validation is essential to verify the physiological and biochemical responses under agronomic conditions.

The insights gained provide a theoretical foundation for precision Cu fertilization and targeted browning control strategies. Given their low cost and compatibility with current agricultural practices, these findings hold strong potential for large-scale application in potato cultivation and processing industries, contributing to improved productivity and reduced postharvest losses.

## 5. Conclusions

Copper plays a concentration-dependent role in potato growth and quality formation, exerting dual regulatory effects on polyphenol oxidase (PPO) activity and amino and organic acid metabolism. An optimal copper concentration (0.157 mmol·L^−1^) enhances chlorophyll accumulation, strengthens photosynthetic efficiency and starch metabolism, and consequently improves tuber yield and quality. In contrast, excessive copper (≥0.235 mmol·L^−1^) induces oxidative stress, disrupts enzymatic homeostasis, and intensifies phenolic oxidation, leading to severe browning and reduced tuber quality. The identified copper threshold offers a scientific basis for precise micronutrient regulation and sustainable potato production. Integrating this threshold into fertigation systems could further enhance yield and quality, reduce postharvest browning in fresh-cut potatoes, and minimize chemical input, thereby contributing to green and efficient potato production.

## Figures and Tables

**Table 1:** Experimental Setup of CuSO_4_ Concentration Gradients.

Treatment	CuSO_4_ (mmol·L^−1^)
CK	0
T1	0.078
T2	0.157
T3	0.235
T4	0.313

**Table 2:** Effects of different concentrations of CuSO_4_ on potato plant growth and yield. Values followed by different lowercase letters indicate significant differences at *p* < 0.05.

Variety	Treatment	Plant Height/cm	Stem Thick/mm	Number of Tubers per Plant/n	Yield per Plant/g	Chl a + b (mg·g^−1^)
Xisen No. 6	CK	32.93 ± 0.71 ^d^	2.20 ± 0.10 ^d^	1.40 ± 0.10 ^b^	10.10 ± 0.07 ^e^	3.49 ± 0.09 ^a^
	T1	34.37 ± 0.67 ^bc^	3.70 ± 0.10 ^b^	1.57 ± 0.15 ^ab^	13.47 ± 0.01 ^c^	3.51 ± 0.18 ^a^
	T2	36.20 ± 0.92 ^a^	3.97 ± 0.12 ^a^	2.41 ± 0.11 ^ab^	21.93 ± 0.02 ^a^	3.63 ± 0.13 ^a^
	T3	35.43 ± 0.78 ^ab^	3.80 ± 0.10 ^ab^	2.50 ± 0.10 ^a^	12.53 ± 0.01 ^d^	2.88 ± 0.16 ^b^
	T4	33.53 ± 0.21 ^cd^	3.37 ± 0.12 ^c^	1.57 ± 0.15 ^ab^	16.33 ± 0.07 ^b^	2.54 ± 0.07 ^c^

**Table 3:** Effects of different concentrations of CuSO_4_ on the color parameters and browning index of potato tubers. Values followed by different lowercase letters indicate significant differences at *p* < 0.05.

Treatment	L*	a*	b*	BI
CK	66.63 ± 0.09 ^a^	1.45 ± 0.02 ^d^	13.97 ± 0.02 ^e^	24.34 ± 0.02 ^e^
T1	63.75 ± 0.02 ^b^	2.05 ± 0.03 ^b^	14.52 ± 0.06 ^d^	27.36 ± 0.03 ^d^
T2	62.91 ± 0.04 ^c^	2.35 ± 0.02 ^c^	14.83 ± 0.04 ^c^	28.75 ± 0.05 ^c^
T3	61.46 ± 0.03 ^d^	3.05 ± 0.03 ^a^	15.34 ± 0.04 ^b^	31.43 ± 0.08 ^b^
T4	61.05 ± 0.02 ^e^	3.07 ± 0.02 ^a^	20.83 ± 0.05 ^a^	44.02 ± 0.14 ^a^

## Data Availability

The original data presented in the study are included in the article; further inquiries can be directed to the corresponding authors.
